# Aeroallergen Der p 2 induces apoptosis of bronchial epithelial BEAS-2B cells via activation of both intrinsic and extrinsic pathway

**DOI:** 10.1186/s13578-015-0063-5

**Published:** 2015-12-21

**Authors:** Chun-Hsiang Lin, Yu-Chuan Hong, Shao-Hsuan Kao

**Affiliations:** Institute of Biochemistry, Microbiology and Immunology, College of Medicine, Chung Shan Medical University, No.110, Sec. 1, Jianguo N. Road, Taichung, 402 Taiwan; Clinical Laboratory, Chung-Shan Medical University Hospital, Taichung, Taiwan

## Abstract

**Background:**

Excessive apoptosis of airway epithelium is reported to induce airway remodeling and inhibited airway epithelium repair is highly associated with development of asthma and chronic obstructive pulmonary disease. Der p 2 is a major allergen derived from *Dermatophagoides pteronyssinus* and commonly causes airway hypersensitiveness and asthma; however, the connection between Der p 2 and epithelial apoptosis remains unclear. This study was aimed to explore whether Der p 2 induces apoptosis of airway epithelial cells and the underlying mechanisms.

**Results:**

Our results showed that recombinant Der p 2 (rDP2) inhibited cell growth and induced apoptosis of human bronchial epithelial cell BEAS-2B. Further investigation revealed that rDP2 increased intracellular reactive oxygen species, level of cytosolic cytochrome c and cleavage of caspase-9 and caspase-3. rDP2 also induced activation of p38 mitogen-activated protein kinase (P38) and c-Jun N-terminal kinase (JNK), and triggered proapoptotic signals including decrease of Bcl-2, increase of Bax and Bak, and upregulation of Fas and Fas ligand. In parallel, rDP2 inhibited glycogen synthase kinase 3beta and consequently enhanced degradation of cellular (FADD-like IL-1β-converting enzyme)-inhibitory protein (c-FLIP). Involvement of toll-like receptor (TLR)2 in rDP2-induced apoptosis was also demonstrated using specific small inhibitory RNA.

**Conclusions:**

Our findings indicate that rDP2 suppresses cell growth and trigger apoptosis of BEAS-2B cells, which may attribute to induction of both intrinsic and extrinsic pathway via TLR2 and P38/JNK signaling and c-FLIP degradation. It suggests that Der p 2 may aggravate respiratory disorders through enhancement of apoptosis and the consequent airway injury.

## Background

Respiratory epithelium is a primary physical barrier which prevents invasion of pathogens and environmental factors such as allergens and air pollutants. In patients with chronic obstructive pulmonary disease and asthma, the epithelial barrier exhibits detachment of columnar ciliated cells, presence of epithelial cell aggregates in sputum, increased permeability to allergens and disturbed expression of the junction molecule at sites of epithelial detachment [1]. These structural changes and functional disorders of airway epithelium are highly associated with increased cell apoptosis and enhanced interactions between the epithelial and immune and/or somatic cells underneath, which putatively induce airway remodeling and irreversible airway damages [2, 3]. In addition, it is evident that level of epithelial damage is correlated with degree of severity of airway hyperresponsiveness [4].

House dust mite (HDM) is one of the most important sources of indoor allergens and has been known as a predominant causative of respiratory disorders such as airway hypersensitiveness, asthma and exacerbation of lung function. HDM derived major allergens are categorized into two groups, proteolytic group-1 and non-proteolytic group-2 on the basis of IgE affinity [5]. Recently, non-proteolytic group-2 HDM allergens including Der p 2 and Der f 2 have received increased interest in the role in activating immune response in asthma patients. Der p 2 has been demonstrated to share not only structural homology but also functional similarity with MD-2 protein that confers responsiveness to lipopolysaccharide in association with toll-like receptor (TLR) 4 [6]. It has also been reported that Der p 2 aggravates respiratory airway disorder by induction of inflammatory cytokines and up-regulation of intercellular adhesion molecule-1 [7]. However, association between Der p 2 and epithelial apoptosis has been rarely explored.

Apoptosis of airway epithelial cells plays a pivotal role in pathogenesis of chronic respiratory disorders [8]. The increase in loss of epithelial integrity in the airway of asthmatics has been suggested as an imbalance between proliferation and apoptosis of epithelial cell, and the resultant decreased adhesion of the epithelial cells to the basement contributes to the epithelial layer shedding in airway [9]. Previous studies have also implicated modulation of cell survival through apoptosis in the pathogenesis of chronic airway diseases [10, 11]. Although apoptosis is postulated as a critical cellular process in the development and progression of chronic airway diseases, influences of aeroallergens on epithelial apoptosis and the underlying signaling cascades remain sketchy. In the present study, we aimed to investigate effects of Der p 2 on airway epithelial cells with emphasis on apoptosis and the underlying mechanisms.

## Methods

### Expression and purification of recombinant Der p 2 (rDP2)

rDP2 was expressed in a pGEX-T vector with the N-terminal glutathione S-transferase (GST) moiety followed by Der p 2 in *E. coli* and purified using glutathione chromatography (Sigma-Aldrich, St. Louis, MO, USA) according to manufacturer’s instructions. Control protein GST was expressed and purified as same as rDP2. After filtrated with 0.22-μm sterile filter (Millipore, Bedford, MA), the purified proteins were quantitated using BCA protein assay kit (Pierce Biotechnology, Rockford, IL, USA) according to the manufacturer’s instructions and used for the following treatments.

### Cell culture and treatments

Immortalized, nontumorigenic human bronchial epithelial cell line BEAS-2B was obtained from ATCC (CRL-2503) and cultured in complete growth medium consisting of RPMI1640 supplemented with 10 % fetal bovine serum (FBS). Cells were maintained at 37 ℃ in a humidified atmosphere with 5 % CO_2_ and subcultured according to resources’ instructions.

For protein expression, cells at a density of 5 × 10^5^/mL were incubated with rDP2 at serial concentrations for 24 h. For kinase signaling analysis, cells at a density of 1 × 10^6^/mL were incubated with rDP2 (20 μg/mL) for 30 min. Inhibition of p38 mitogen-activated protein kinase (P38) or c-Jun N-terminal kinase (JNK) was performed by pretreating cells with 10 μM SB203580 or SP600125 (Sigma-Aldrich) for 30 min, respectively. Inhibition of Akt activation was performed by pretreating cells with wortmannin at 200 nM for 30 min.

### Cell viability assay

Cell viability was determined using 3-(4,5-cimethylthiazol-2-yl)-2,5-diphenyl tetrazolium bromide (MTT) assay. Cells were seeded at a density of 4 × 10^4^ cells/well in a 24-well plate and cultured for 24 h. Then, the cells were treated with rDP2 at 5, 10, 20 and 40 μg/mL for 24 h. After the treatments, cells were washed with phosphate-buffered saline (PBS), and then incubated with 5 mg/mL MTT solution for 4 h. After removing the supernatant, isopropanol was added to solubilize the resulting formazan. Absorbance at 563 nm was measured for cell viability. Three independent experiments were performed for statistical analysis.

### Cell cycle distribution analysis

Cells were seeded in 6-well plates at a density of 1 × 10^5^ cells and treated with varying concentrations of rDP2 (10, 20, 40 μg/mL), GST (40 μg/mL) or vehicle for 24 h. After incubation, cells were trypsinized, washed twice with ice-cold PBS, and fixed overnight in 70 % ethanol at 4 ℃. Cells were then resuspended in PBS and treated with 1 mg/mL of RNase A and 1 mg/mL of propidium iodide in PBS for 45 min. Cell-cycle distribution was evaluated using flow cytometer (FACS Calibur, version 2.0, BD Biosciences, Franklin Lakes, NJ, USA) using CellQuest software. Quantitative data were obtained from three independent experiments.

### Detection of apoptotic cells

The apoptotic cells were detected and quantitated by using terminal deoxynucleotidyl transferase dUTP nick end labeling (TUNEL) system (Promega, Madison, WI, USA) and propidium iodide (PI)/annexin V method (R&D Systems, Minneapolis, MN, USA) according to the manufacturer’s instructions. For TUNEL system, 4′,6-diamidino-2-phenylindole (DAPI) was used for nuclei staining. Signal detection was performed using a fluorescence microscopy (LSM 710, Carl-Zeiss, Oberkochen, Germany). The index of apoptosis was the average of the percentages of positive nuclei including apoptotic bodies per total number of nuclei from five random observation fields at 200× magnitudes. For PI/annexin V system, cells were washed with PBS and resuspended in medium containing GST (40 μg/mL) or rDP2 (5 and 20 μg/mL). Cells staining with PI and binding fluorescein isothiocyanate (FITC)-labeled annexin V were determined by flow cytometer (FACS Calibur, version 2.0, BD Biosciences, Franklin Lakes, NJ, USA) using CellQuest software. Quantitative data were obtained from three independent experiments.

### Intracellular ROS determination

Production of intracellular reactive oxygen species (ROS) was determined by spectrofluorometrical method using 2′,7′-dihydrodichlorofluorescein diacetate (DCFH-DA) assay with modification [24]. DCFH-DA diffuses through the cell membrane and is enzymatically hydrolyzed by intracellular esterases to the nonfluorescent DCFH, which can be rapidly oxidized to the highly fluorescent DCF, the fluorescent product, in the presence of ROS. After exposure to GST or rDP2, DCFH-DA was added to the culture plates at a final concentration of 5 μM and incubated for 40 min at 37 ℃ in darkness. DCF fluorescence intensity was detected with emission wavelength at 530 nm and excitation wavelength at 485 nm using a SpectraMax Plus microplate reader (Molecular Devices Corporation, Sunnyvale, CA, USA). The values were expressed as the mean absorbance normalized to the ratio of control value.

### Subcellular fractionation

Cells were washed with PBS and incubated with lysis buffer (10 mM HEPES, pH 7.6; containing 15 mM KCl, 2 mM MgCl_2_, 0.1 mM EDTA, 1 mM dithiothreitol, 0.05 % Igepal CA-630 and 1 mM PMSF, 1 mM Na_3_VO_4_, 50 mM NaF, 10 μg/mL leupeptin/aprotinin) for 10 min. Cell lysates were collected by a centrifugation at 2500 g for 10 min at 4 ℃. The supernatant containing the cytosol was further centrifuged at 20,000g for 15 min at 4 ℃, namely cytosolic fraction. The pellets containing nuclei were washed with PBS, resuspended in nuclear buffer (25 mM HEPES, pH 7.6, 0.1 % Igepal CA-630, 1 M KCl, 0.1 mM EDTA, 1 mM PMSF, 1 mM Na_3_VO_4_, 2 mM NaF, 10 μg/mL leupeptin/aprotinin), and centrifuged at 10,000g for 15 min at 4 ℃. The resulting supernatants were collected, namely nuclear fraction.

### Immunoblotting

Cells were collected and lysed for protein extraction and the followed immunoblotting as previously described [12]. Phosphorylation and level of protein was demonstrated by using antibodies against human cellular proteins, including caspase-3, caspase-9, caspase-8, cytochrome c, Poly (ADP-ribose) polymerase (PARP), Bcl-2, Bak, Bax, Fas, FasL, phosphorylated P38 (p-P38), total P38, phosphorylated JNK (p-JNK), total JNK, phosphorylated glycogen synthase kinase 3beta (GSK3β) (Ser9), cellular FLICE inhibitory protein (c-FLIP), and α-tubulin (Cell signaling, Beverly, MA). Detection of antigen–antibody complex was performed by using ECL reagent (Millipore, Bedford, MA, USA) and luminescence image system (LAS-4000 mini; Fujifilm, Tokyo, Japan). Semi-quantitation of reacted signals was determined using Multi Gauge software version 2.2 (FujiFilm, Tokyo, Japan). Three independent analyses were performed for statistical analysis.

### RNA extraction and quantitative real-time PCR (qPCR)

Total RNA extraction was performed by using RNeasy mini kit (Qiagen, Hilden, Germany). cDNA was synthesized from total RNA by reverse transcription using RevertAid First Strand cDNA Synthesis Kit (Thermo Fisher Scientific Inc, Waltham, MA). qPCR was performed using the ABI PRISM 7700 sequence detection system (Applied Biosystems, Foster City, CA). For mRNA quantitation, FastStart Universal SYBR Green Master (Roche Applied Science, Mannheim, Germany) was used for Taqman PCR. Relative gene expressions were calculated by using the 2^−ΔΔCt^ method [2]. Expression of GADPH gene was used an internal endogenous control. All qPCR experiments were performed in duplicates for each sample. The correct size of the PCR products was confirmed by agarose gel electrophoresis.

### Inhibition of TLR2 expression with small interfering RNA (siRNA)

To specifically inhibit TLR2 expression, a combination of three different siRNAs (3 target sequence for TLR2; UUC UCA UCU CAC AAA AUU G, CUU GUG ACC GCA AUG GUA U, and UCU UUA UGU CAC UAG UUA U) was used for transfection. Transfection was performed according to the manufacture’s instruction. Briefly, cells were seeded in a 96-well plate at 2 × 10^4^ cells/well and incubated at 37 ℃ and 5 % CO_2_ for 16 h. The siRNA solution was prepared in RNase-free buffered solution and the final concentration of each siRNA was 1 μM per well (Dharmacon^®^ AccellTM siRNA reagents, Thermo scientific, Hudson, NH, USA). After 72 h, the transfected cells were treated with rDP2 for 24 h.

### Statistical analysis

Data were expressed as means ± SEMs of the three independent experiments. Statistical significance analysis was determined by using 1-way ANOVA followed by Dunnett for multiple comparisons with the control or the impaired 2-tailed Student *t* test. The differences were considered significant for *p* values less than 0.05.

## Results

### rDP2 reduced viability and induced apoptosis of BEAS-2B cell

**Fig. 1:**
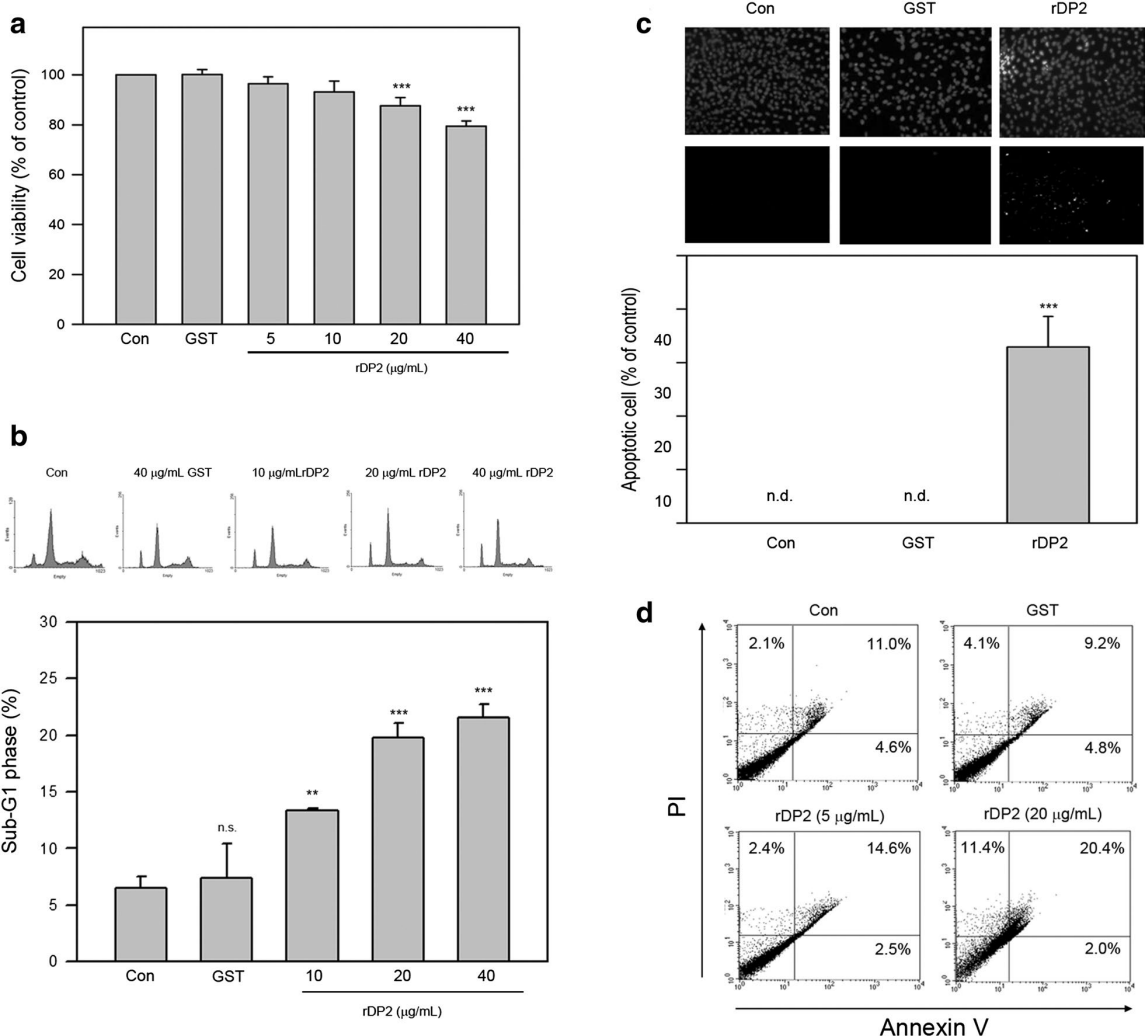
rDP2 reduced cell viability and induced cellular apoptosis of BEAS-2B cells. Cells were treated with rDP2 at indicated concentrations for 24 h, and then the cells were subjected to **a** cell viability assay, **b** cell cycle assay by flow cytometric analysis, **c** TUNEL assay, or **d** PI/annexin V staining with flow cytometric analysis. Three independent experiments were performed for statistical analysis. **p < 0.01 and ***p < 0.005 as compared to GST treatment (40 μg/mL, 24 h)

Effects of rDP2 on epithelial cell viability were investigated using MTT assay. As shown in Fig. 1a, 24 h-rDP2 treatments reduced cell viability of BEAS-2B in a dose-dependent manner and the viability was decreased up to 79.4 ± 0.7 % of control in response to 40 μg/mL rDP2 (*P* < 0.005 as compared to GST treatment). To confirm the rDP2-induced cell death, cell cycle distribution analysis was performed and the results revealed that rDP2 dose-dependently elevated sub-G1 phase up to 22.4 ± 1.3 % (*P* < 0.005 as compared to GST treatment). In parallel, the GST treatment slightly increased sub-G1 phase to 7.2 ± 3.3 % (*P* = 0.273 as compared to negative control) (Fig. 1b). In addition, TUNEL assay showed that apoptotic cell ratio was increased to 24.1 ± 4.2 % of control in response to rDP2 treatment (40 μg/mL) (*P* < 0.005 as compared to GST treatment), and no TUNEL-positive cell was detected in the control and the GST treatment (Fig. 1c). PI/annexin V staining was also used for detection of apoptotic cell. As shown in Fig. 1d, rDP2 significantly increased apoptotic cell ratio from 14.6 (5 μg/mL) to 20.4 % (20 μg/mL) as compared to GST (9.2 %). These findings revealed that rDP2 triggered apoptotic cell death and consequently reduced the cell viability of BEAS-2B.

### rDP2 induced increase of intracellular ROS and activation of intrinsic apoptosis cascades in BEAS-2B cell

**Fig. 2:**
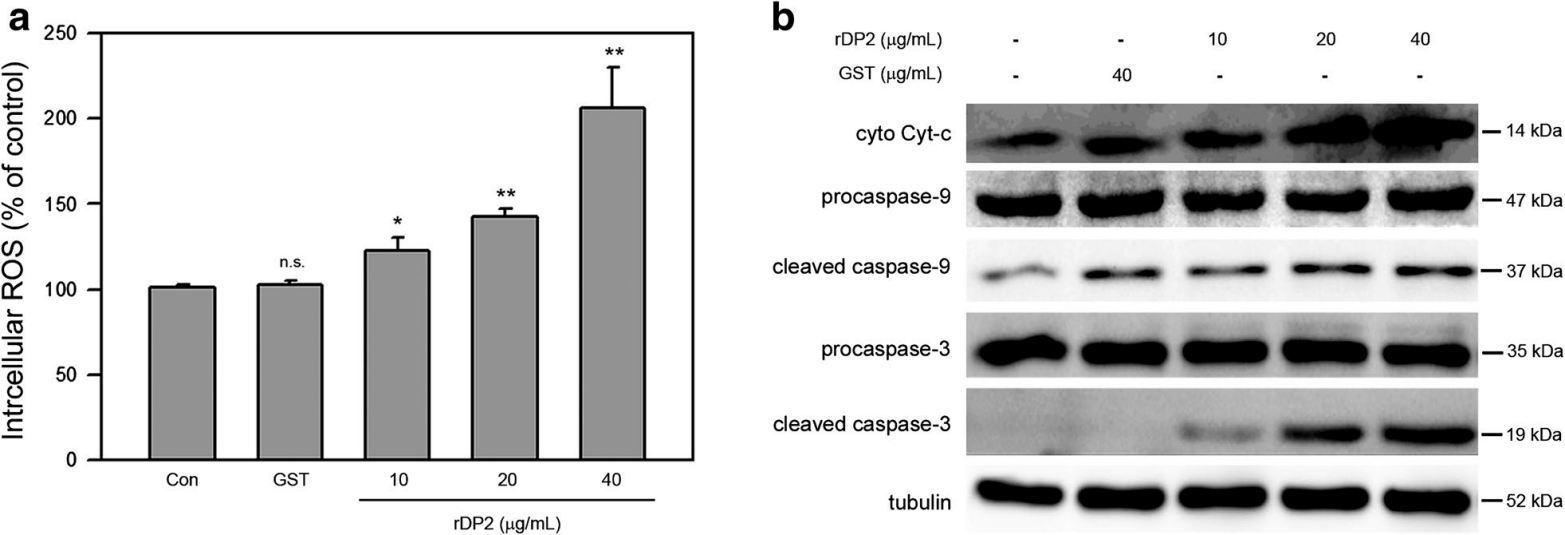
rDP2 increased intracellular ROS and cytosolic cytochrome c and induced activation of caspase-9 and caspase-3 in BEAS-2B cell. Cells were treated with rDP2 at indicated concentrations for 24 h, and then the cells were subjected to **a** intracellular ROS assay or **b** immunoblotting for indicated proteins. Three independent experiments were performed for statistical analysis. *p < 0.05 and **p < 0.01 as compared to GST treatment (40 μg/mL, 24 h). Signals of tubulin were used as internal control

ROS is commonly assumed as an important stress factor involving in pathogenesis of allergic airway diseases and activation of mitochondrial pathway [13]. To investigate whether rDP2 induced ROS, intracellular ROS level was then determined. As shown in Fig. 2a, rDP2 increased level of intracellular ROS in a dose-dependent manner and the intracellular ROS level was elevated up to 208.6 ± 28.7 % of negative control in response to 40 μg/mL rDP2 treatment (*P* < 0.01 as compared to GST treatment) (Fig. 2a). In addition, level of cytosolic cytochrome c was significantly increased in BEAS-2B cells treated with rDP2. In parallel to increased cytosolic cytochrome c, our results showed that rDP2 triggered activation of caspase-9 and the downstream effector caspase-3 (Fig. 2b). In contrast to rDP2, GST treatment neither significantly altered level of intracellular ROS and cytosolic cytochrome c nor triggered activation of caspase-9 in BEAS-2B cells (Fig. 2). Taken together, these findings showed that rDP2 elevated level of intracellular ROS, cytosolic cytochrome c and activation of mitochondrial pathway.

### rDP2 triggered intrinsic apoptotic signaling in BEAS-2B cell via activation of P38 and JNK

**Fig. 3:**
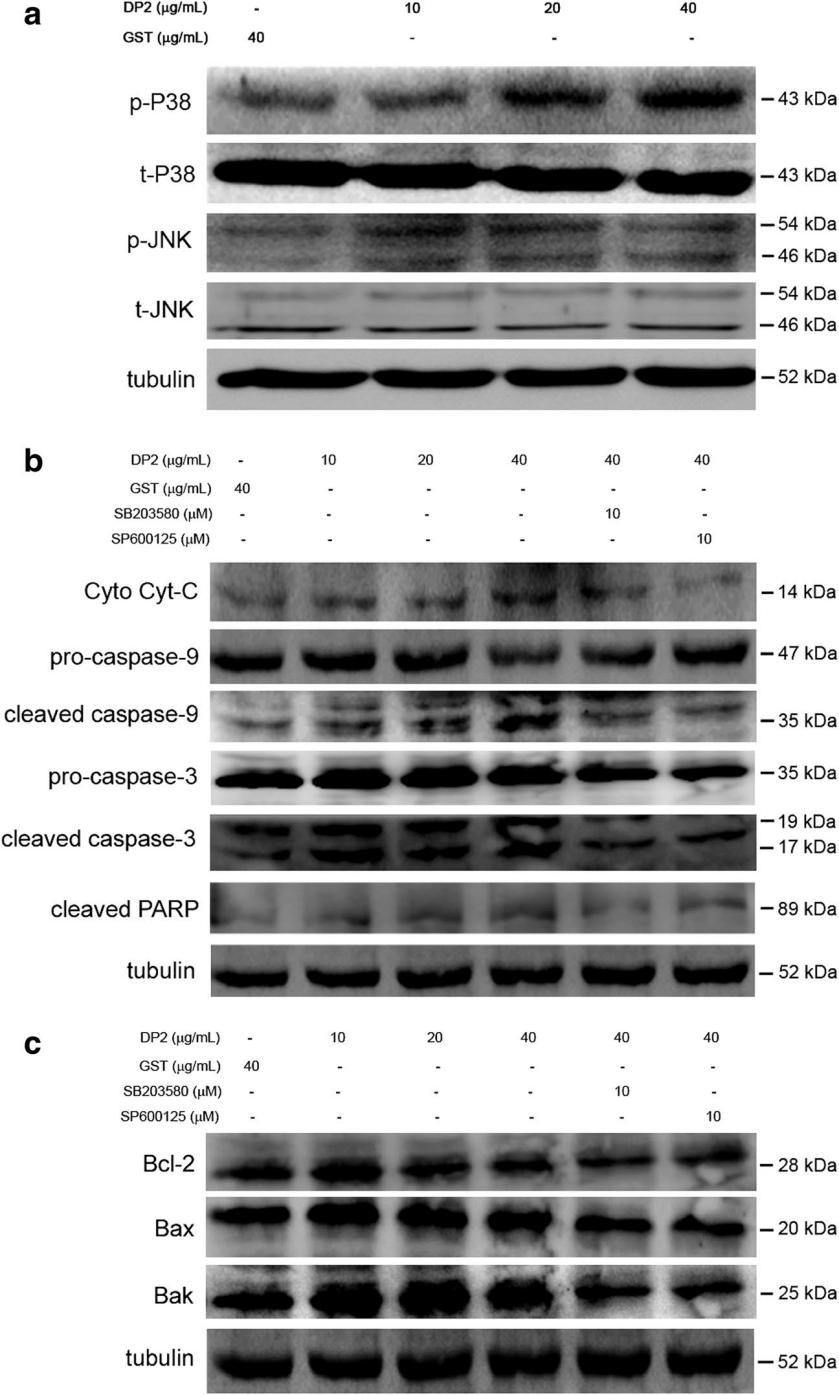
Involvement of P38 and JNK in activation of caspases and imbalance of pro-apoptotic components in BEAS-2B cell stimulated with rDP2. Cells were treated with rDP2 at indicated concentrations for 30 min (**a**) or pretreated with indicated inhibitors for 2 h and then with rDP2 at indicated concentrations 24 h (**b**, **c**), and then the cells were subjected to immunoblotting for indicated proteins. Signals of tubulin were used as internal control

P38 and JNK have been reported to play important roles in mitochondrial pathway and ROS generation [14, 15]. Therefore, involvement of P38 and JNK activation in rDP2-triggered mitochondrial pathway was investigated. As shown in Fig. 3a, rDP2, but not GST, significantly induced phosphorylation of P38 and JNK. In addition, inhibition of P38 and JNK by specific inhibitor SB203580 and SP600125 diminished the DP-induced cleavage of caspase-9, caspase-3 and PARP (Fig. 3b). In parallel, levels of anti-apoptotic Bcl-2 and pro-apoptotic Bax and Bak were decreased and increased respectively in response to rDP2 (Fig. 3c). The increase of Bax and Bak by rDP2 was reversed by pretreatment of SB203580 and SP600125; however, the decrease of Bcl-2 by rDP2 was insignificantly affected by the inhibitors (Fig. 3c). These results revealed that rDP2 induced activation of P38 and JNK that involved in mitochondrial apoptotic signals in BEAS-2B cell in response to rDP2.

### rDP2 promoted expression of Fas and FasL in BEAS-2B cell through P38 and JNK activation

**Fig. 4:**
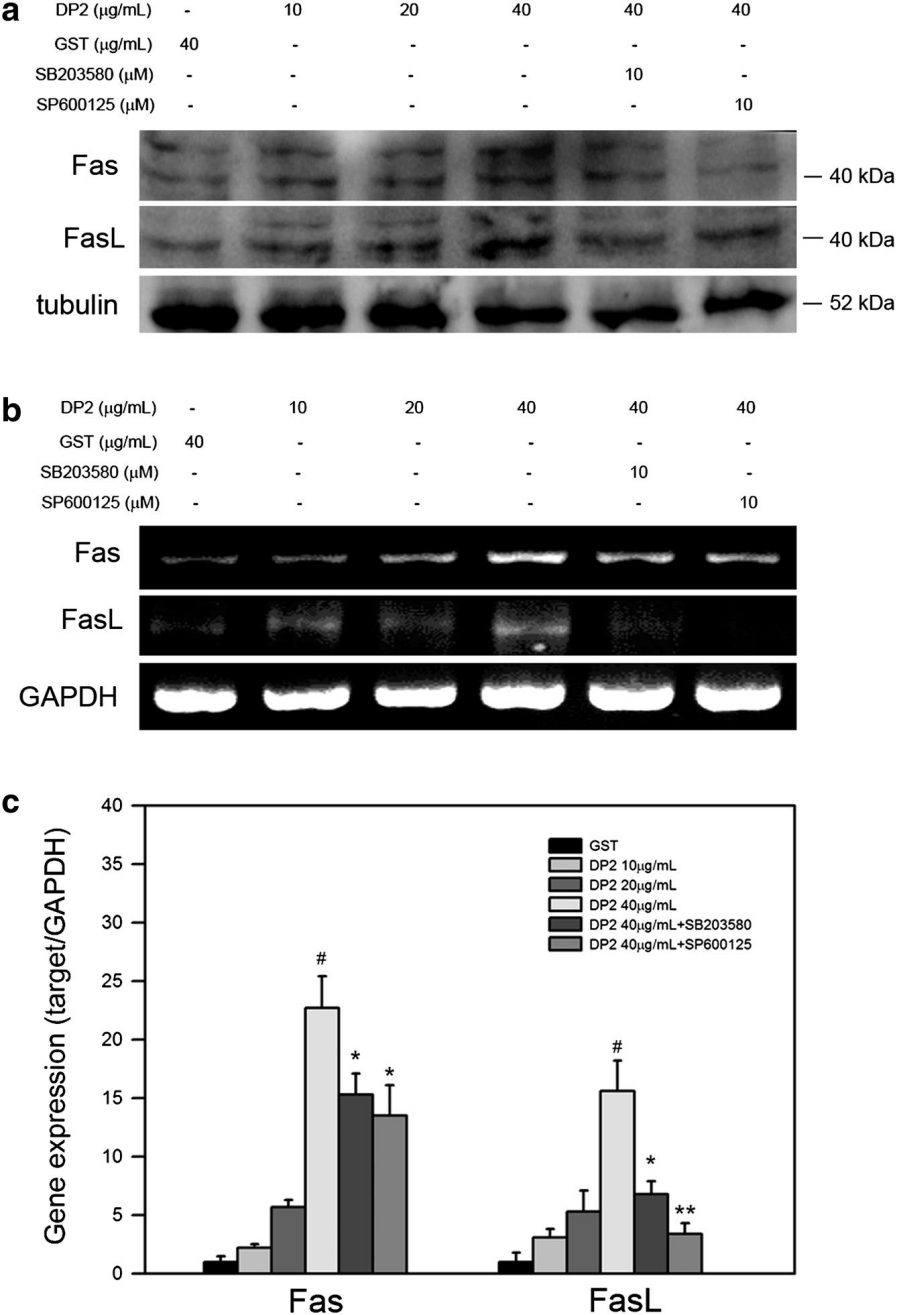
Involvement of P38 and JNK in rDP2-mediated upregulation of Fas and FasL in BEAS-2B cell. Cells were pretreated with or without indicated inhibitors, and then treated with rDP2 at indicated concentrations for 24 h (**a**) or 6 h (**b**, **c**). The treated cells were subjected to **a** immunoblotting, **b** RT-PCR, or **c** qPCR for Fas and FasL expression, respectively. Signals of tubulin and GAPDH were used as internal control for immunoblotting and RT-PCR, respectively. Three independent experiments were performed for statistical analysis. #*p* < 0.05 as compared to GST treatment. *p < 0.05 and **p < 0.01 as compared to 40 μg/mL rDP2 treatment

In addition to mitochondrial pathway, P38 and JNK have also been reported to associate with extrinsic pathway via regulating Fas and Fas ligand (FasL) expression [16]. Therefore, whether rDP2 affects expression of Fas and FasL is investigated. As shown in Fig. 4a, rDP2 increased protein level of FasL and Fas, and inhibition of P38 and JNK activation by SB203580 and SP600125 diminished the increase of Fas and FasL in BEAS-2B cell stimulated by rDP2. Similar results were also observed in mRNA expression of Fas and FasL determining by RT-PCR and qPCR, respectively (Fig. 4b, c). Collectively, these findings showed that rDP2 upregulated both protein and mRNA expression of Fas and FasL in BEAS-2B cells via P38 and JNK activation.

### rDP2 enhanced caspase-8 activation via Akt/GSK3β-mediated down-regulation of c-FLIP in BEAS-2B cell

**Fig. 5:**
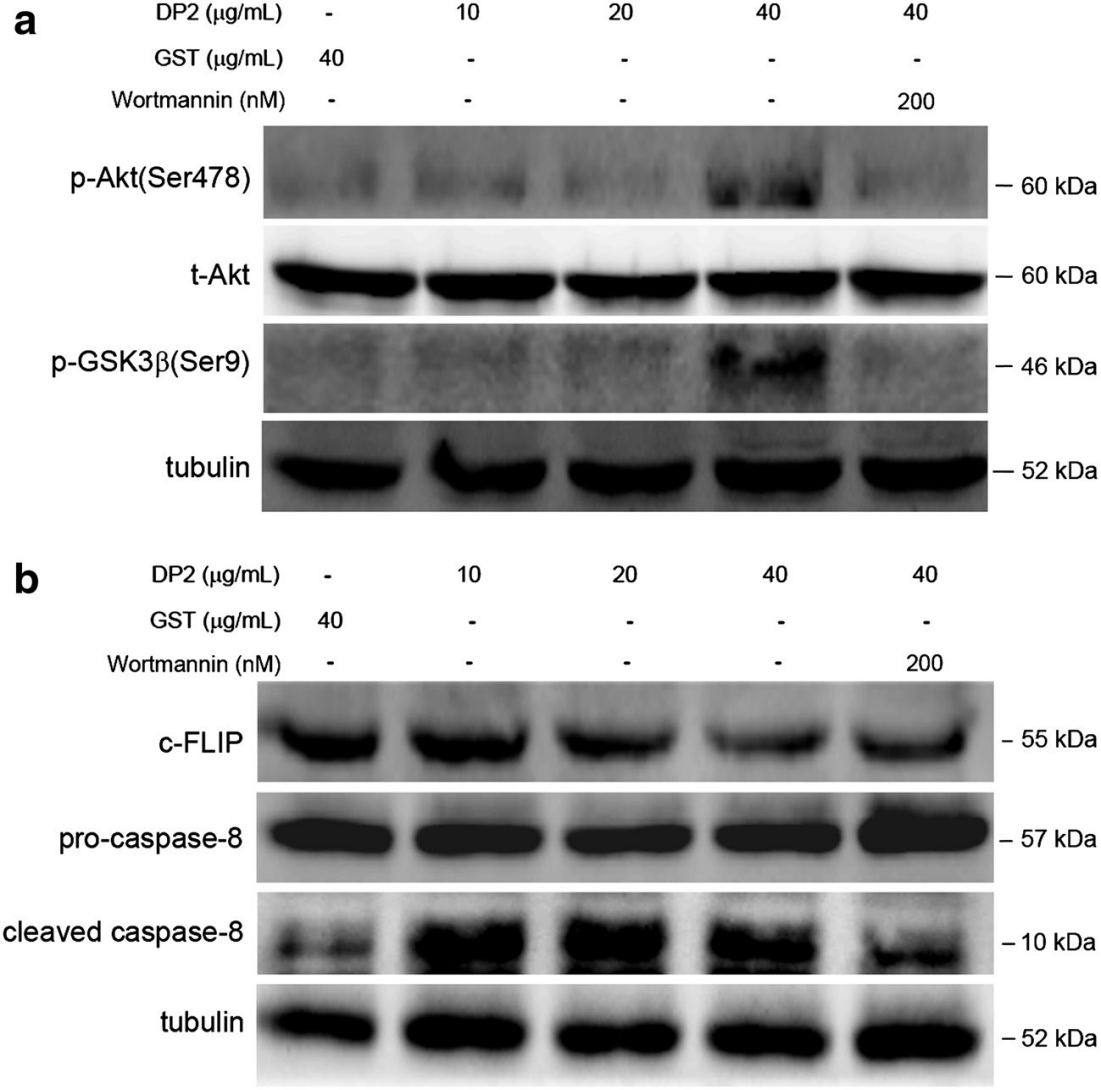
Involvement of Akt/GSK3β and c-FLIP in activation of caspases-8 in BEAS-2B cell treated with rDP2. Cells were pretreated with or without wortmannin for 2 h, and then treated with rDP2 at indicated concentrations for 30 min. The treated cells were subjected to immunoblotting for **a** Akt/GSK3β phosphorylation, and **b** c-FLIP/caspase-8 cleavage. Signals of tubulin were used as internal control

GSK3β is regarded as an important mediator that controls level of c-FLIP, a caspase-8 inhibitor [17]. Our previous study demonstrates that rDP2 inhibits GSK3β through Akt-mediated phosphorylation at serine 9 in human lung carcinoma A549 cell [12]. Therefore, whether rDP2 induces phosphorylation of GSK3β (Ser9) and consequently alters level of c-FLIP in BEAS-2B cell is further investigated. As shown in Fig. 5a, phosphorylated GSK3β (Ser9), consistent with phosphorylated Akt (Ser478), was increased in BEAS-2B cells treated with rDP2, and the increase of phosphorylated GSK3β (Ser9) was significantly diminished by pretreatment of PI3K inhibitor wortmannin. Further results revealed that level of c-FLIP was dose-dependently decreased in BEAS-2B cells in response to rDP2. In parallel to decrease of c-FILP level, cleaved caspase-8 level was significantly increased (Fig. 5b). Notably, the rDP2-induced decrease of c-FLIP and increase of cleaved caspase-8 was restored by pretreatment of wortmannin. These results showed that rDP2 enhanced caspase-8 activation attributing to Akt/GSK3β-mediated down-regulation of c-FLIP in BEAS-2B cell.

### Involvement of TLR2 signaling in DP2-induced apoptosis of BEAS-2B cell

**Fig. 6:**
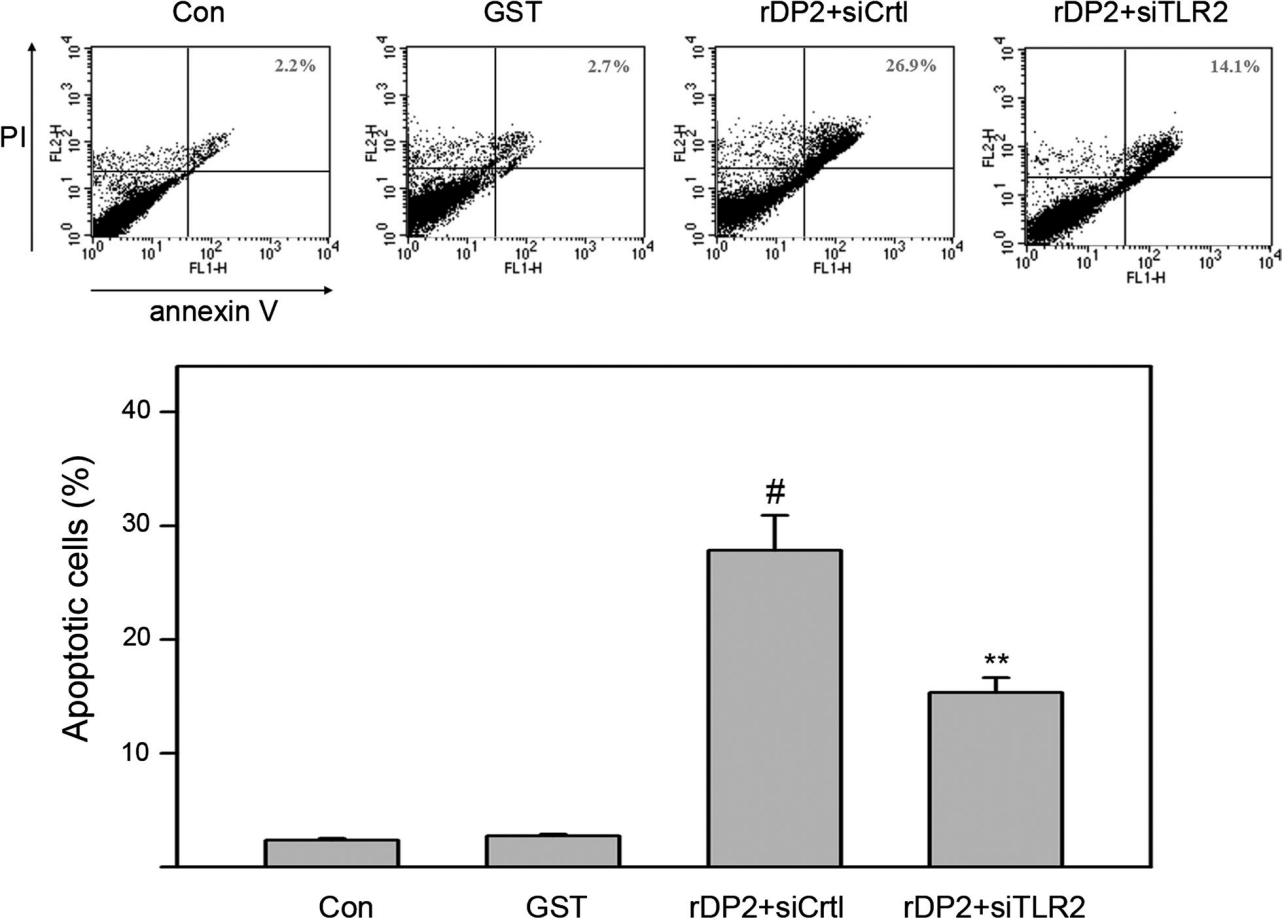
Involvement of TLR2 in rDP2-induced apoptosis of BEAS-2B. Cells were transfected with siRNA against TLR2 or control siRNA (siCrtl) for 72 h, treated with rDP2 or GST for 24 h, and then was subjected to PI/annexin V staining with flow cytometric analysis. Three independent experiments were performed for statistical analysis. #*p* < 0.05 as compared to GST treatment. **p < 0.01 as compared to 40 μg/mL rDP2 + siCrtl treatment

Der p 2 is known to activate TLR2/MyD88/NF-κB signaling and triggers inflammatory responses [18]. Thus, the role of TLR2 in Der p 2-induced apoptosis is investigated. As shown in Fig. 6, silencing TLR2 expression by specific siRNA decreased apoptotic cell ratio from 27.0 ± 2.7 % to 14.1 ± 1.6 % in response to rDP2 treatments (P < 0.05). These findings showed that TLR2 signaling may partially involve in rDP2-induced apoptosis of BEAS-2B cells.

## Discussion

Apoptosis is commonly observed in alveolar epithelial cells in pulmonary fibrosis, a condition that predisposes to severe asthma [18]. Asthmatic bronchial epithelium is characteristically damaged with loss of columnar epithelial cells that is highly relative to unregulated apoptosis [19]. The present study indicates that non-proteolytic aeroallergen rDP2 reduces viability of BEAS-2B cell via inducing apoptosis, suggesting that rDP2 not only evokes immune responses but also directly causes damage of airway epithelium. Previous studies have indicated roles for ROS in the pathology of asthma both in terms of increased stress and decreased antioxidant protection [20]. In addition, airway responses have been shown to correlate with oxidant generation by eosinophils after antigen challenge in vivo [21] and neutrophil superoxide generation correlates with bronchial hyperreactivity [22]. Our results demonstrate that rDP2 promotes intracellular ROS level, which may contribute to the increase of Bax/Bcl-2 ratio and the cytochrome c release in BEAS-2B cell. These findings provide evidences that rDP2 may contributes to airway hyperreactivity via enhancing intracellular ROS and the consequent pathogenic and pro-apoptotic signaling in epithelial cells.

Cellular apoptosis is mediated by a fine balance of pro- and anti-apoptotic proteins in cells. Particularly, the mitochondrial apoptosis pathway is regulated by early translocation of anti-apoptotic (Bcl-2 and Bcl-XL) and pro-apoptotic (Bak and Bax) members of the Bcl-2 family of proteins to or from mitochondria [23], which modulate the release of pro-apoptotic components such as cytochrome c that activate the apoptosome, effecter caspases and DNases resulting in DNA fragmentation and cleaved PARP. Furthermore, the presence of cleaved PARP (p85) has been reported as an important marker of early apoptosis in asthmatic bronchial epithelium [19]. Our results reveal that rDP2 decreases Bcl-2 but increases Bax and Bak level in BEAS-2B cells, as well as increases p85 PARP, indicating that rDP2 triggers intrinsic apoptosis of human bronchial epithelial cells attributing to imbalance of pro-apoptotic and anti-apoptotic members.

A recent study reports that *Dermatophagoides pteronyssinus* (DP) extract can inhibit apoptosis of human neutrophil [24]. The DP extract, generally, consists of more than ten types of allergens that have been identified and characterized [5]. Among the identified DP allergens, group 1 and group 3 is known to possess protease activity, and the others are non-proteolytic [5]. In this study, our findings indicate that rDP2 evokes apoptosis of epithelial cell BEAS-2B. It suggests that the discrepancy may result from different cell types in response to multiple allergens and single allergen. However, it is interesting that mite allergens can prolong the neutrophil by inhibiting its apoptosis and meanwhile, induce the apoptosis of airway epithelial cell, which synergically enhances inflammatory response and airway remodeling that further exacerbates the airway injury. Further investigation is needed to clarify the underlying mechanism.

MAPK signaling cascades were revealed in a myriad of fundamental cellular processes including proliferation, motility, stress reaction and apoptosis. Among MAPKs, JNK and P38 activation are generally pro-apoptotic in response to stress and cellular damage [25, 26]. JNK/P38 activation has been reported to contribute to hyperphosphorylation of c-Jun and promote transcription of FasL, leading to apoptosis of ovarian carcinoma cell [27]. In addition, active P38 phosphorylates Bcl-xL and Bcl-2 and prevents the accumulation of these anti-apoptotic components within the mitochondria, contributing to loss of mitochondrial membrane potential and the release of cytochrome c [28]. P38 is also known to mediate Fas-induced mitochondrial death pathway in CD8+ T cells [28]. Interestingly, our results reveal that rDP2 up-regulates both mRNA expression and protein level of Fas and FasL via JNK and P38 activation, indicating that rDP2 not only triggers intrinsic pathway but also reinforces extrinsic apoptosis via up-regulation of death-inducing receptor and its ligand through JNK/P38 pathway.

c-FLIP is a death effector domain (DED)-containing family member that inhibits one of the most proximal steps of death receptor (DR)-mediated apoptosis. After being recruited to the death-induced signaling complex (DISC), c-FLIP suppresses pro-caspase-8 activation and inhibits DR-mediated apoptosis via different mechanisms [29, 30]. A recent study demonstrates that a linkage between GSK3 inhibition and c-FLIP down-regulation, thus highlighting a new mechanism by which GSK3 modulates the extrinsic apoptotic pathway [31]. Similar with the previous findings, the present study reveals that rDP2 inhibits GSK3β by Akt-mediated phosphorylation at serine-9, consequently down-regulating c-FLIP and enhancing extrinsic apoptotic pathway.

Der p 2 has been reported to induce inflammatory responses via activation of TLR2/4 and the downstream MyD88/NF-kB pathway [6, 7, 32]. The capability of TLR activation links Der p 2 to be regarded as an innate immunity activator. A very recent study shows that Der p 2 can be internalized by epithelial cell that promotes TLR-mediated proinflammatory signaling [33]. These findings demonstrates that TLRs play a crucial role in Der p 2-triggered cellular signaling and inflammatory responses. Similarly, our results indicates that TLR2 partially involves in rDP2-induced epithelial apoptosis. However, the TLR2-independent apoptotic signaling in response to Der p 2 needs further investigation.

In summary, this study shows a novel mechanism by which rDP2 induces mitochondrial pathway and promotes extrinsic pathway by up-regulation of Fas/FasL and down-regulation of c-FLIP through Akt-dependent phosphorylation of GSK3β (Ser9). Through this study, we are able to show, for the first time, that the non-proteolytic aeroallergen rDP2 directly triggers apoptosis of airway epithelial cell via both intrinsic and extrinsic pathway and highlights the role of JNK/P38 and TLR2 signaling in the rDP2-induced apoptosis, thus providing a new reasonable explanation for how rDP2 exacerbates airway damage in chronic respiratory diseases.

## Abbreviations

rDP2: recombinant Der p 2
DAPI: 4′,6-diamidino-2-phenylindole
DCFH-DA: 2′,7′-dihydrodichlorofluorescein diacetate
FasL: Fas ligand
GSK3β: glycogen synthase kinase 3beta
GST: glutathione S-transferase
JNK: c-Jun N-terminal kinase
P38: p38 mitogen-activated protein kinase
MTT: 3-(4,5-cimethylthiazol-2-yl)-2,5-diphenyl tetrazolium bromide
ROS: reactive oxygen species
c-FLIP: cellular (FADD-like IL-1β-converting enzyme)-inhibitory protein
TLR2: toll-like receptor 2
TUNEL: terminal deoxynucleotidyl transferase dUTP nick end labeling
